# The circadian and photoperiodic clock of the pea aphid

**DOI:** 10.1007/s00359-023-01660-8

**Published:** 2023-07-24

**Authors:** Francesca Sara Colizzi, David Martínez-Torres, Charlotte Helfrich-Förster

**Affiliations:** 1 University of Würzburg, Neurobiology and Genetics, Biocenter, Am Hubland, 97074 Würzburg, Germany; 2 Institut de Biologia Integrativa de Sistemes, Parc Cientific Universitat de València, C/ Catedrático José Beltrán nº 2, 46980 Paterna,, València Spain

**Keywords:** Parthenogenetic, Sexual reproduction, Insulin-like peptide, Cryptochrome, Timeless, Pigment-dispersing factor, Dampened clock

## Abstract

The pea aphid, *Acyrthosiphon pisum,* is a paradigmatic photoperiodic species that exhibits a remarkable annual life cycle, which is tightly coupled to the seasonal changes in day length. During spring and summer, characterised by longer days, aphid populations consist exclusively of viviparous females that reproduce parthenogenetically. When autumn comes and the days shorten, aphids switch their reproductive mode and generate males and oviparous sexual females, which mate and produce cold-resistant eggs that overwinter and survive the unfavourable season. While the photoperiodic responses have been well described, the nature of the timing mechanisms which underlie day length discrimination are still not completely understood. Experiments from the 1960’s suggested that aphids rely on an ‘hourglass’ clock measuring the elapsed time during the dark night by accumulating a biochemical factor, which reaches a critical threshold at a certain night length and triggers the switch in reproduction mode. However, the photoperiodic responses of aphids can also be attributed to a strongly dampened circadian clock. Recent studies have uncovered the molecular components and the location of the circadian clock in the brain of the pea aphid and revealed that it is well connected to the neurohormonal system controlling aphid reproduction. We provide an overview of the putative mechanisms of photoperiodic control in aphids, from the photoreceptors involved in this process to the circadian clock and the neuroendocrine system.

## Introduction

**Fig. 1:**
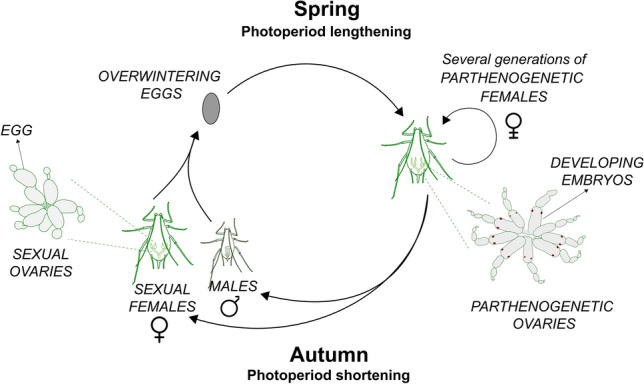
The annual life cycle of the pea aphid consists of several parthenogenetic generations during spring and summer followed by one sexual generation in autumn that produces overwintering eggs. Details see text

Photoperiodism is the ability to perceive day length (photoperiod) as an anticipatory cue of seasonal changes and to respond with appropriate physiological and behavioural adjustments (Bradshaw and Holzapfel 2007). Aphids (Hemiptera: Aphididae) were the first animals described as photoperiodic (Marcovitch 1924). In temperate regions of the planet, they adapt to seasonal changes with a remarkable life cycle (Fig. 1). In spring and summer, which are characterised by long days, aphid populations consist exclusively of viviparous females that reproduce parthenogenetically (i. e. clonally). Aphid parthenogenetic ovaries contain embryos that, at advanced stages, themselves already have ovaries with newly developing embryos, ensuring rapid and efficient reproduction. When autumn comes and the nights lengthen, aphids switch their reproductive mode and generate males and oviparous sexual females. This photoperiodic switch is maternal meaning that a factor deriving from the neurosecretory system of the mother’s brain determines the fate of the unborn embryos as viviparous parthenogenetic females or as sexual males/females (Lees 1964; Steel and Lees 1977; Steel 1978). Adult sexual morphs mate and the females produce cold-resistant eggs that overwinter and survive the unfavourable season. In the next spring, those eggs hatch and the newly born nymphs initiate a new series of viviparous parthenogenetic female generations that succeed one another until the next autumn. Thus, induction of the sexual generation in aphids is equivalent to activation of diapause in other insects, which is also usually regulated by photoperiod (Saunders et al. 1990; Nakamura and Hodkova 1998; Saunders 2002).

Most importantly, in early spring when photoperiods are as short as in autumn, the newly hatched aphids do not switch back to sexual reproduction but continue to be parthenogenetic (Markovitch 1924). Bonnemaison (1949, 1972) found that the aphids remain unresponsive to the photoperiod for a certain time after hatching and postulated the presence of a ‘fundatrix-factor’ that prevents initiation of the sexual phase. Lees (1960) suggested the existence of an ‘interval timer’ determining this insensitivity to short days, and showed that it lasted 25 to 45 days in *Megoura viciae* at 20 °C. More recently, Matsuda et al. (2017, 2020a,b) characterised the physiological nature of the interval timer in detail in *Acyrthosiphon pisum.* These authors noted that animals did not respond to short days for 70–90 days after hatching from winter eggs and named the timer responsible ‘seasonal timer’ to avoid confusion with the ‘hourglass’ mechanism for measuring day length in autumn, sometimes called also ‘interval timer’ (see below). The mechanisms of the seasonal timer have been recently reviewed in detail (Matsuda 2023a) and are not discussed here.

Our paper reviews the mechanisms of day length measurement that determine the photoperiodic switch from parthenogenetic to sexual reproduction in autumn.

## Putative mechanisms of day length measurement

### Theoretical components of photoperiodism—hourglass versus circadian clock

**Fig. 2:**
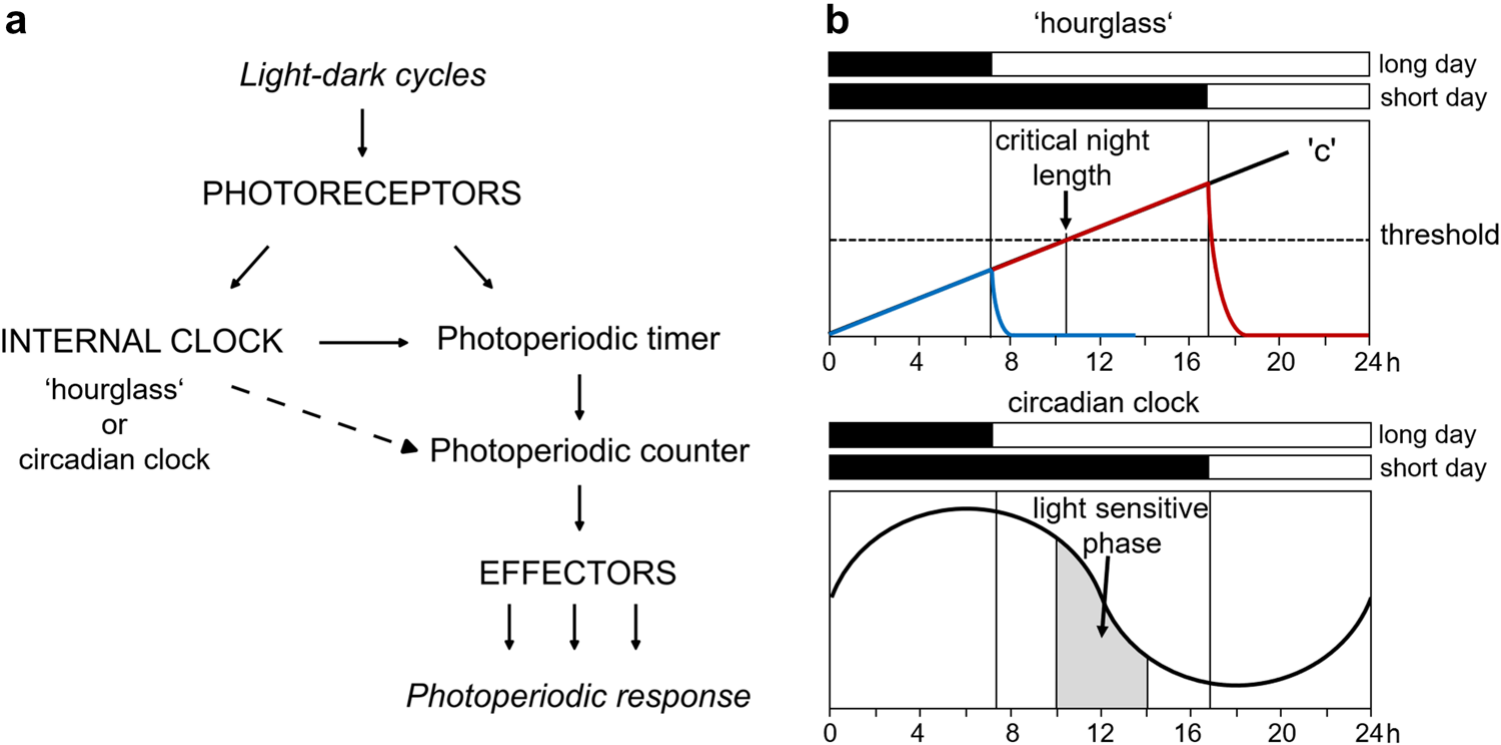
Components of the photoperiodic system. **a** Photoreceptors sense light from the external light–dark cycles. The light signals are received by the photoperiodic timer and an internal clock that can either be an ‘hourglass’ or a circadian clock. **b** In case of the ‘hourglass’, a signal is transmitted to the photoperiodic timer when substance ‘c’ exceeds a critical threshold (at the critical night length) and, in case of the circadian clock, when light falls into the photosensitive phase of the circadian clock. The photoperiodic counter counts the number of days at which this happens and activates the effector after a certain number of inductive days have been counted

Photoperiodism encompasses several components that are summarized in Fig. 2 and reviewed in Saunders (2002). One or more types of photoreceptors perceive light and transmit this information to the “PHOTOPERIODIC TIMER”, which controls the photoperiodic response by comparing the information from the photoreceptors with that coming from an internal reference timer, also called “INTERNAL CLOCK”. The internal clock does also receive input from photoreceptors. The photoperiodic timer determines whether the photoperiod falls below a certain threshold to initiate the photoperiodic response. In other words, it determines whether the duration of the night exceeds a certain length—the ‘critical night length’. Subsequently, the PHOTOPERIODIC COUNTER counts the days at which the ‘critical night length’ is exceeded and activates the EFFECTORS after a certain number of inductive days have been counted. The effectors (for example animal neurohormones) then trigger the photoperiodic response, e. g. an overwintering response (diapause) or the sexual reproduction in aphids.

Two views on the nature of the internal clock have been the subject of discussion. It can be either a simple interval timer (‘hourglass’) or a circadian clock (Vaz Nunes and Saunders 1999; Saunders 2010). In the hourglass-system, night length is indicated by a steadily accumulating light-sensitive factor ‘c’ and this information is sent to the photoperiodic timer (Fig. 2b). The photoperiodic timer, in turn, sends signals to the photoperiodic counter when this factor ‘c’ exceeds a critical threshold. Thus, the photoperiodic timer determines the critical threshold of factor ‘c’ and consequently also the ‘critical night length’. However, when light from the photoreceptors interrupts the night and degrades ‘c’ before it reaches its critical threshold no signal will be sent to the photoperiodic counter. This happens under long days and means that the animals remain in their ‘summer state’. Such an ‘hourglass’ mechanism was proposed for the vetch aphid *Megoura viciae* (Lees 1966) and the spider mite *Tetranychus urticae* (Veerman and Vaz Nunes 1987).

Alternatively, the circadian clock can serve as time reference as was proposed by Bünning (1936) many years ago and strongly supported and further evolved by Pittendrigh (1972). Bünning developed his model originally for the timing of plant flowering in spring and later extended to the overwintering response (diapause) of the lepidopteran *Pieris brassicae* (Bünning and Joerrens 1960). The model assumes a rhythmic light sensitivity of the circadian clock that continues even in constant darkness (Fig. 2b). When light interacts with the light-sensitive phase, induction or inhibition of the photoperiodic response follows through the photoperiodic timer, counter and effectors as described above. While the circadian clock is self-sustained, the ‘hourglass’ clock depends completely on the light–dark cycles. It is reset by light of a certain duration and starts anew during the dark period.

**Fig. 3:**
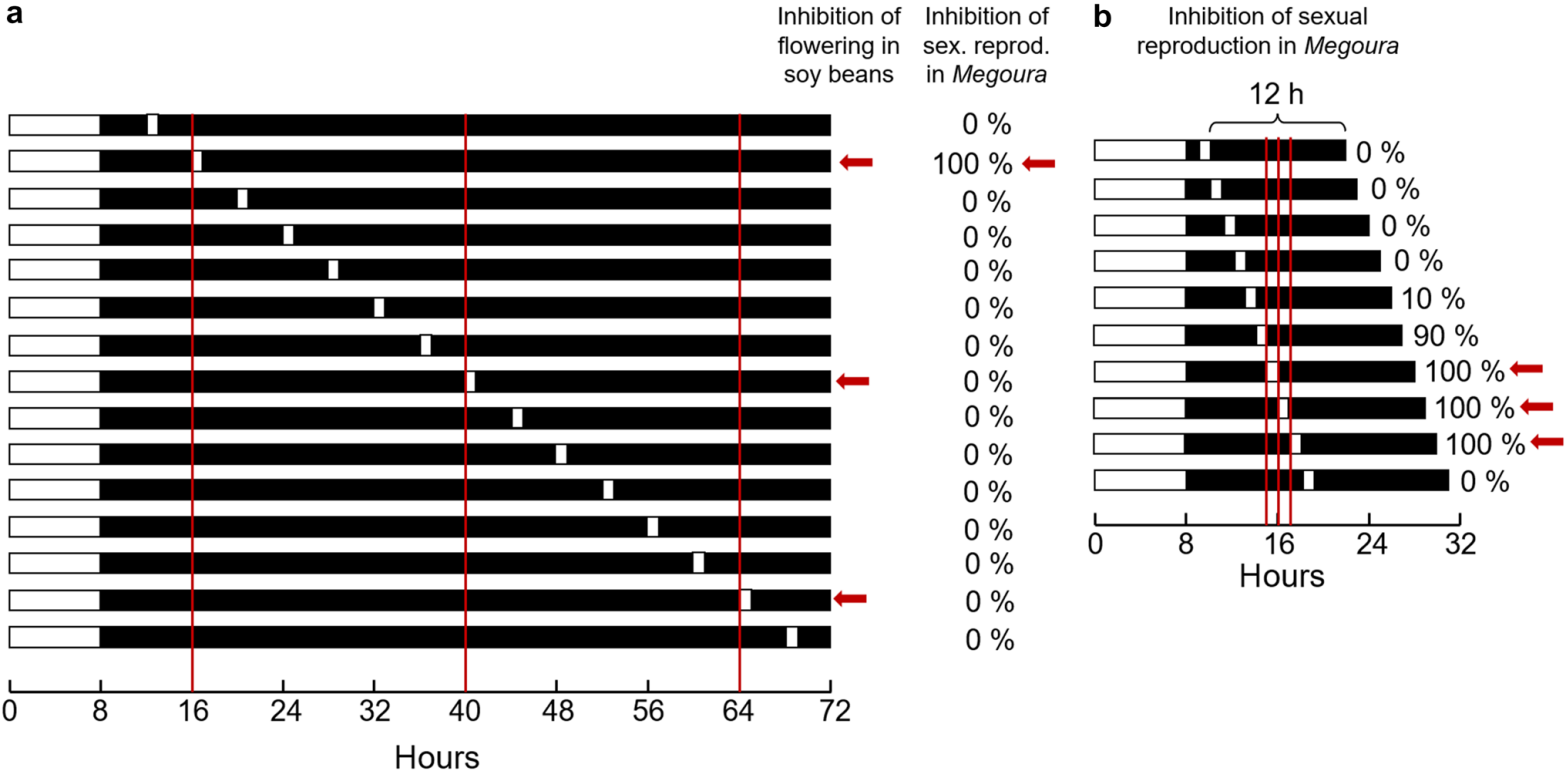
Inhibition of sexual reproduction (production of oviparous offspring) by 1 h light pulses delivered during long nights in aphids that were held in a short photoperiod of 8 h, which normally leads to sexual reproduction. **a** A long night of 64 h was interrupted by one light pulse occurring at different times in different experiments. In soybeans such light pulses inhibit flowering every ~ 24 h (red arrows and red vertical lines) suggesting a rhythmical light sensitivity of the underlying clock. In *Megoura viciae*, the inhibiting effect of the light pulse was only found during the ‘first cycle’, 16 h after lights-on of the main photoperiod. **b** Similar night-interrupting light pulses with the difference that the dark period after the light pulse was kept constant at 12 h and that the light pulses were delivered in a narrower time frame. Once more, sexual reproduction was inhibited around the time of 16 h after lights-on of the main photoperiod. Red arrows highlight 100% successful inhibition. Figure redrawn and modified after Lees (Lees 1966)

Bünning’s hypothesis can be tested by night-interrupting light pulses, which have been performed in several plants and insects and are known as Nanda-Hamner and Bünsow experiments (Nanda and Hamner 1958; Bünsow 1960; see also Bradshaw et al. this issue; Saunders this issue). For instance, short-day plants (flowering is induced by short days) such as the Biloxi soybean, can be kept under short flowering-inducing photoperiods of 8 h followed by a very long night of 64 h, which is interrupted at different times by 1 h light pulses (Fig. 3a). These 1-h light pulses are expected to hit the photosensitive phase of the circadian clock every ~ 24 h and to inhibit flowering. Indeed, this was observed for Biloxi soybeans: flowering was inhibited by light pulses occurring at 16 h, 40 h and 64 h after lights-on of the main light period and this result was regarded as positive Nanda-Hamner response (Hamner 1960) (Fig. 3a).

### Aphids appear to use a highly dampened circadian clock as time reference

The same experiments were performed in the vetch aphid, *Megoura viciae*, but they yielded different results (Lees 1966). Only the light pulse occurring at 16 h after lights-on (equals 8 h after lights-off) inhibited the production of sexual oviparous females; all other light pulses could not interrupt the sexual reproduction (Fig. 3a). This result is hard to reconcile with Bünning’s hypothesis, nor with an alternative model offered by Pittendrigh (1972) that also relies on the circadian clock as a basis (Saunders 1978; Saunders this issue for further explanation). However, it is also difficult to explain by a pure ‘hourglass’ model because the dark period following the 1-h light pulse occurring 16 h after lights-on is very long (56 h) and clearly exceeds the critical night length of 9.75 h needed in *Megoura* to induce sexual reproduction, and yet it did not result in sexual reproduction.

**Fig. 4:**
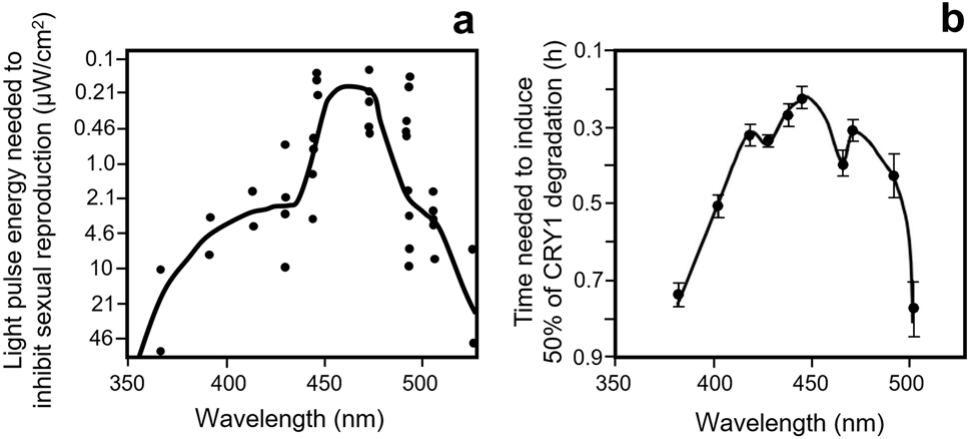
Action spectra for photoperiodic responses in the vetch aphid and for cryptochrome 1 (CRY1, d-CRY) degradation. **a** Semilogarithmic plot showing the inhibiting responses of 1-h monochromatic light pulses on sexual reproduction administered during the most sensitive phase in the early night. The intensity of the response is given by the percentage of aphids that produced viviparous progeny and the curve is drawn at the ~ 50% response level (modified and replotted from Lees (1966)). Note that light pulses in the late night have a higher proportion of red-light sensitivity, suggesting that opsins contribute to the photoperiodic responses of Megoura viciae (Lees 1981) **b** Semilogarithmic plot showing CRY1 degradation after exposure of living flies to monochromatic light of 17 µmol/m2 s2. The amount of CRY1 was determined on western blots of head extracts in comparison with non-irradiated control flies, and the curve shows the time needed to degrade CRY1 to 50% of the controls (modified from Hoang et al. 2008)

Similarly, another experiment performed by Lees (1966) is hard to reconcile with an ‘hourglass’. In this experiment, the aphids were again subjected to oviparous offspring-inducing short photoperiods (8 h) followed by a long night interrupted by a 1-h light pulse, but the night length after the light pulse was kept constant at 12 h of darkness (Fig. 3b). Again, the 12 h of darkness after the night-interrupting light pulse should always lead to sexual reproduction if a simple ‘hourglass’ clock is involved, but it did not. Only when the duration of the dark period before the light pulse was longer than 5 h was sexual reproduction completely or almost completely inhibited. In fact, 100% inhibition was found when the light pulse occurred 7 to 9 h after lights-off (Fig. 3b). This coincides with the light pulse at 8 h after lights-off (or 16 h after lights-on) of the previous experiment, which also yielded to 100% inhibition of sexual reproduction (Fig. 3a). No inhibition was again found when the light pulse occurred later, e. g. 10 h after lights-off, even though such a late light pulse divides the night into 2 long nights (each longer than the 9.75 h critical night lengths needed for sexual reproduction). Thus, it appears logical that only aphids producing sexual oviparous offspring were found. The latter experiments show additionally that the photoinducible phase of the oscillator occurs in the second half of the night (between 6 and 10 h after lights-off of the main light period) (Fig. 3b). This time point coincides with “point B” in the diapause-regulating photoinducible phase shown for several insects (see Fig. 4 in Saunders this issue).

Although the results of the just mentioned experiments, do not support Bünning’s hypothesis of a rhythmically re-occurring photosensitive phase, which is expected from an underlying circadian clock, they do well support the existence of a dampened circadian clock that would show a single photosensitive phase ~ 16 h after lights-on of the previous light–dark cycle, before its oscillations would dampen out. This conclusion is in line with results from modelling studies. Saunders and Lewis (1987) were able to simulate positive and negative Nanda-Hamner and Bünsow phenotypes by simply adjusting the degree of oscillator dampening in the model, suggesting that the dampening tendency of the oscillator is a primary cause of the positive and negative responses. Hardie and Vaz Nunez (2001) modelled the photoperiodic clock of English and Scottish clones of the black bean aphid, *Aphis fabae* and found that it can be explained by a slowly damping long-night system and a rapidly damping short-night system. Even the ‘classical’ example of an ‘hourglass’ type of photoperiodic clock—the *Megoura viciae* clock, was shown to have a circadian basis (Vaz Nunes and Hardie 1993).

Evidence for circadian behaviour has been shown in different aphid species. A circadian rhythm in host-finding behaviour has been suggested in potato aphids (*Macrosiphum euphorbiae*) (Narayandas et al. 2006). Marked rhythms in larviposition and fresh-weight increase, independent of their host rhythm, were reported in *Myzus persicae* (Hodgson and Lane 1981). In the greenbug (*Schizaphis graminum*) and in the green citrus aphid (*Aphis spiraecola*), the onset of the release of the sex pheromone by oviparous females has also been shown to be governed by a circadian clock (Eisenbach and Mittler 1980; Jeon et al. 2003). Most interestingly, recent results with pea aphids (*Acyrthosiphon pisum*) indicate that these aphids show circadian rhythms in locomotion, honeydew excretion and hatching from eggs and, most relevant, they show that these rhythms are highly dampened as they disappear quickly after exposure to constant darkness (Joschinski et al. 2016; Beer et al. 2017; Matsuda 2023b). Similarly, circadian expression of pea aphid clock genes *per, tim* and *Cry2* (see below) was shown to be quickly dampened under constant darkness (Barberà et al. 2017; 2022). Dampened weak circadian clocks have the advantage that they can easily phase-shift and thus quickly adapt to changing photoperiods (see Vaze et al. this issue).

Although weak circadian clocks cannot keep rhythmicity under constant conditions, they cycle with high amplitude under light–dark cycles (Bertolini et al. 2019) and are thus perfectly suited to serve as time reference for the photoperiodic timer. This property could explain why several animals living at high latitudes have weak circadian clocks and accept becoming arrhythmic under constant conditions (Pflüger and Neumann 1971; Lankinen and Riihimaa 1992; van Oort et al. 2005; Stokkan et al. 2007; Kauranen et al. 2012; Menegazzi et al. 2017; Beauchamp et al. 2018; Hazlerigg and Tyler 2019).

## Nature and location of the aphid photoperiodic receptors, internal clock and effectors

### Pioneering studies

Classic studies from the 1960s and 1970s have shown that the photoperiod is perceived by the brain of the mother aphid and the photoperiodic message is then transmitted to the ovaries where the embryos develop (Lees 1964; Steel and Lees 1977; Steel 1978). By precisely illuminating different parts of the body of vetch aphids kept under short photoperiods (long nights), Lees (1964) was able to show that induction of sexuality was only abolished when the dorsal part of the mother’s head was illuminated, suggesting that the dorsal part of the brain harbours the photopigment that senses day length, while the eyes did not appear to be involved at all. By conducting action spectra for the photoperiodic response, Lees (1966; 1981) furthermore showed that the photopigment involved in photoperiodism is most sensitive to blue light (450–470 µm) with several shoulders extending to the near UV (365 µm) but also to the red (Fig. 4). This speaks for the involvement of several photoreceptors, putatively a flavin for the responses to short wavelength and opsins for the longer wavelengths. The involvement of opsins in photoperiod perception has been shown by raising insects on a medium deficient in Vitamin A, which is needed for the synthesis of the opsin chromophore retinal, and for normal photoperiodic responses (Saunders 2002; Nelson et al. 2009).

Some years later, Steel (1977) described five groups of neurosecretory cells (NSC groups I to V) in the *protocerebrum* of the vetch aphid. Of these, axons originating from the ten cells in the *pars intercerebralis* (NSC group I; five cells per hemisphere) and axons originating from one cell per hemisphere of NSC group II in the *pars lateralis* formed a bundle leading to the abdomen and most likely to the ovaries. More importantly, the ablation of group I NSCs resulted in the production of sexual offspring in aphids that have been reared under long photoperiods (Steel and Lees 1977). Similarly, ablation of the adjacent areas in the *pars lateralis* had the same effects. This led the authors to hypothesize that NSCs of group I produce a parthenogenesis-promoting factor under long photoperiods, which they called virginoparin. This virginoparin would be transported to the embryos and signal them to develop as parthenogenetic females. Thus, the virginoparin may be the effector of the aphid photoperiodic system. Steel and Lees (1977) also suggested that the *pars lateralis* contains the photoreceptors and the photoperiodic timer that signal to the group I NSCs in the *pars intercerebralis* through synaptic interactions.

### Recent studies on the molecular nature of the photoperiodic effectors

Different transcriptomic approaches identified genes showing differential expression in aphids reared under long and short photoperiods, and were thus considered good candidates to be involved in aphid photoperiodism (Ramos et al. 2003; Cortés et al. 2008; Le Trionnaire et al. 2009, 2012). Once the aphid genome became available (The International Aphid Genomics Consortium 2010), attention was focused on the identification and analysis of the expression of candidate genes to be components of the aphid photoperiodic clock. Among these were genes encoding pea aphid photoreceptors (Collantes-Alegre et al. 2018; Barberà et al. 2022), circadian clock genes (Cortés et al. 2010; Barberà et al. 2017) and different genes encoding putative output factors of the circadian clock, such as those involved in the synthesis of melatonin (Escrivá et al. 2016; Barberà et al. 2020), as well as several genes encoding putative effectors of the photoperiodic timer, such as juvenile hormone (JH) (Ishikawa et al. 2012), prothoracicotropic hormone (PTTH) (Barberà and Martínez-Torres 2017) and various neuropeptides, including insulin-like peptides (Huybrechts et al. 2010), among others.

Melatonin is a well-known hormonal output of the vertebrate circadian clock that is synthesized during the night (Klein 2007). It plays an essential role in the seasonal physiology of vertebrates, providing information on photoperiod (Wehr 1997; Pévet et al. 2006; Arendt 2019). Although a role in insect photoperiodism is not stablished, in one study it was shown that melatonin-fed pea aphids were partially induced to develop sexual morphs under summer-like photoperiod, suggesting a role for this hormone in triggering the seasonal response also in aphids (Gao and Hardie 1997). More recently, the presence of melatonin was demonstrated in the pea aphid (Escrivá et al. 2016) and showed to reach higher levels in aphids reared under long nights than in aphids kept under long days (Barberà et al. 2020). Furthermore, this difference in melatonin content was not observed in aphids of an anholocyclic strain that does not respond to short days. In addition, insect-specific AANAT coding genes (assumed to be rate limiting for melatonin synthesis) showed higher expression under short photoperiod (Barberà et al. 2020).

Similarly, the expression of genes involved in JH biosynthesis and the insulin pathway (an insulin-degrading enzyme and an insulin receptor) was modified by the photoperiod (Le Trionnaire et al. 2009; 2012). While the gene coding for the insulin-degrading enzyme was highly expressed under short-day conditions, the gene coding for the insulin receptor was highly expressed under long day conditions, suggesting a role of insulins during long photoperiods. Finally, Barberà et al. (2019) proposed a putative link between pea aphid insulin-like peptides and the virginoparin, long ago anticipated by Steel and Lees (1977) as an effector promoting parthenogenesis. Of the 10 insulin-like peptide (ILP) coding genes initially identified in the pea aphid (Huybrechts et al. 2010), and recently updated to a total of 7 genes (Huygens et al. 2022), only ILP1 and ILP4 showed higher expression in aphid heads under long photoperiods than under short photoperiods (Barberà et al. 2019), thus fulfilling the required characteristics to be considered virginoparins.

**Fig. 5:**
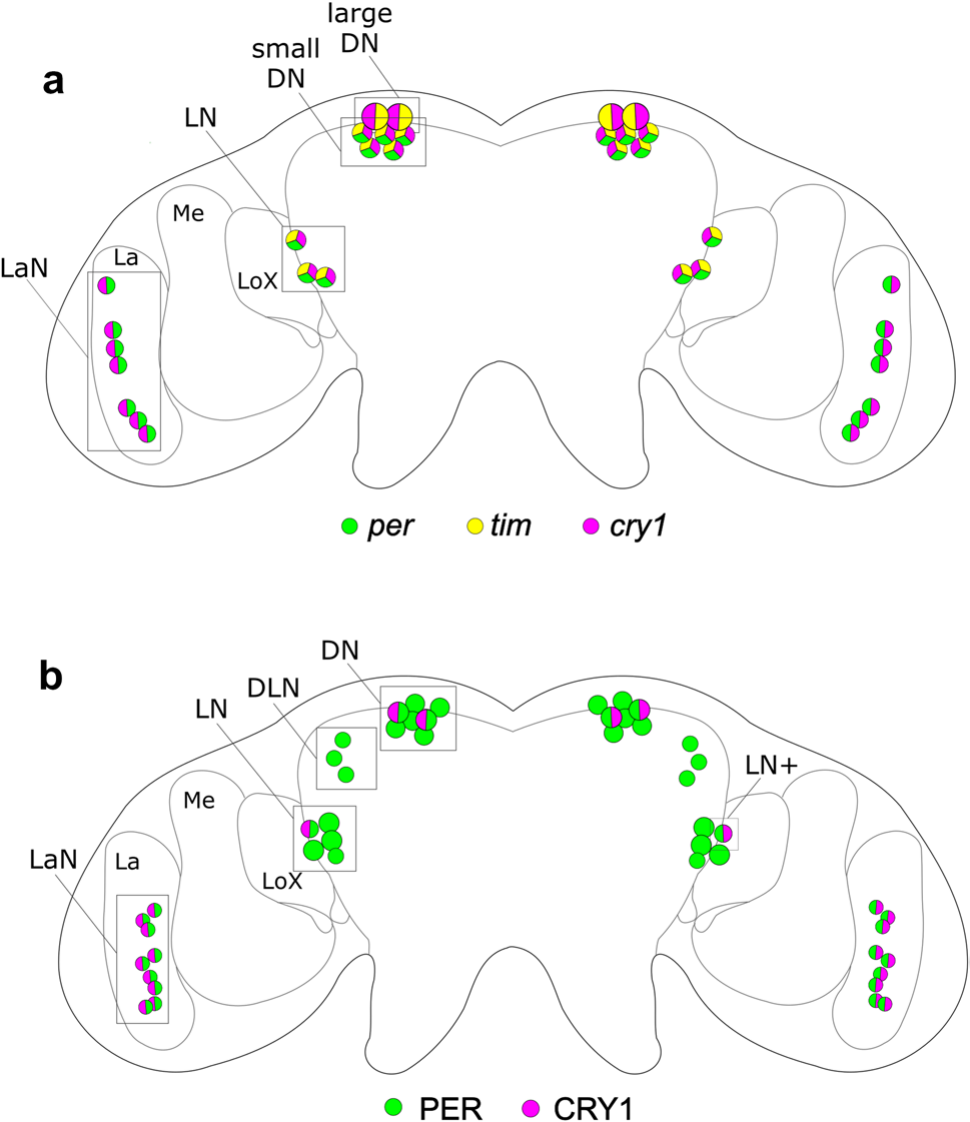
Identification of the clock neurons by location of clock transcripts and proteins. **a** *per*, *tim*, and *cry1* clock transcripts were localised in the lateral and dorsal protocerebrum by *in situ* hybridisation. Additionally, about seven clock neurons were localised in the lamina, but they lack *tim* (Barberà et al. 2017; 2022) Most interestingly, two large neurons in the dorsal protocerebrum co-express *tim* and *cry1*, but appear not to express *per*. **b** The use of antibodies against PER and CRY1 principally confirmed the findings from the *in situ* hybridisation, but revealed slightly more clock cells in the lamina and the lateral protocerebrum including an additional cluster located between the LN and DN (DLN, Dorso Lateral Neurons). Furthermore, the CRY1 antibody labelled only two dorsal cells per hemisphere instead of seven revealed by *in situ* hybridisation. Figure modified after Colizzi et al. (2021)

ILP1 and ILP4 were co-expressed in four cells (per hemisphere) in the *pars intercerebralis*, most likely corresponding to four of the five cells in the NSC group I, originally described by Steel (1977) in *M. viciae* (see above and Fig. 5). These results were strongly supported by an immunohistochemical mapping of ILP4 in the vetch and pea aphid (Cuti et al. 2021). In this study, a newly produced antibody against ILP4 stained four neurosecretory cells (per hemisphere) in the *pars intercerebralis,* which possessed putative dendrites extending into the *pars lateralis* and projected to the *corpora cardiaca*, a neurohormonal release site of brain-derived insect neuropeptides. From there, three nerves travelled further down into the abdomen via three tracts, one median and two laterals. Although no precise site of release has been found, the termination of these nerves near the ovaries is compatible with the proposed direct connection between group I NSCs and the reproductive system (Cuti et al. 2021). Furthermore, the putative dendrites of the ILP4-positive cells in the *pars lateralis* appear perfectly suited to get input from the proposed photoperiodic photopigments traced to this region (Steel and Lees 1977).

ILPs released in the *corpora cardiaca* work also on the glandular JH producing part of the *corpora allata* stimulating JH synthesis (Nässel and Zandawala 2019; Leyria et al 2023). JH is a sesquiterpenoid that controls insect metamorphosis, reproduction and maternal diapause regulation (Mukai et al. 2022). In aphids held under short days, the application of a JH homologue led to the production of asexual offspring, indicating that JH promotes parthenogenetic reproduction (Corbitt and Hardie 1985; Hardie and Lees 1985). Thus, ILPs from the neurons in the *pars intercerebralis* might not only promote parthenogenetic reproduction by acting on the ovaries but also by promoting JH synthesis in the *corpora allata*.

### The aphid circadian clock

Circadian clocks are generally based on molecular transcriptional and translational feedback loops that are interconnected and lead to rhythmic expression of genes (reviewed by Pilorz et al. 2018; Beer and Helfrich-Förster 2020). In *Drosophila melanogaster*, whose clock is the best studied among insects, the basic feedback loop involves the genes *period* (*per*), *timeless* (*tim*), *Clock* (*Clk*), *cycle* (*cyc*), and their respective protein products PER, TIM, CLK, and CYC. CLK and CYC form heterodimers that act as positive transcription factors for *per*, *tim*, and other genes. PER and TIM proteins themselves form heterodimers that enter the nucleus and inhibit their own transcription by binding the CLK/CYC complex. A second feedback loop involving the clock genes *par domain protein 1* (*pdp1*) and *vrille* (*vri*) stabilizes the first one by provoking rhythmic transcription of *Clock*. For synchronization with the external light–dark cycle, the *Drosophila* clock uses photoreceptors in their eyes, as well as the blue-light photopigment Cryptochrome 1 (CRY1, also called *Drosophila* CRY or d-CRY), which is in the neurons of the circadian clock itself (reviewed by Helfrich-Förster 2020). Activation of CRY1 by light leads to the breakdown of TIM, resetting the clock daily.

*A. pisum* was shown to contain in its genome most of the orthologs of the *Drosophila* circadian clock genes, although with some differences. In addition to CRY1, similar to other insects, aphids possess another form of cryptochrome, which is orthologous of the mammal cryptochromes and is called Cryptochrome 2 (CRY2 or mammalian CRY (m-CRY); Zhu et al. 2005; Rubin et al. 2006; Yuan et al. 2007; Cortés et al. 2010). This type of cryptochrome has been shown to be insensitive to light and to take over the role of TIM in the master clock (Kotwica-Rolinska et al. 2019). Aphids belong to those insects that possess both TIM and CRY2. As expected for bona-fide core clock genes *per*, *tim*, *cry2*, *cyc*, *pdp1* and *vri* are rhythmically expressed in aphid heads (Cortés et al. 2010; Barberà et al. 2017). The rhythmic expression of *cyc* is consistent with results from mammals and most other insects, while *cyc* expression in *D. melanogaster* is arrhythmic. The CYC protein of *D. melanogaster* lacks the C-terminal transactivation domain for binding PER, while the latter is present in aphids (Takahata et al. 2000; Chang et al. 2003; Rubin et al. 2006).

Another difference with the *Drosophila* clock system is the absence of the gene *jetlag* in the pea aphid genome (Cortés et al. 2010). The Jetlag protein interacts in a light-dependent way with the clock proteins CRY1 and TIM and is involved in the synchronisation of the clock with the daily light–dark cycle (Koh et al. 2006; Peschel et al. 2009). Furthermore, some clock genes, particularly those that are in the light-input pathway to the clock (including *tim*, *per* and *cry2*), are highly divergent, when compared with other insects, suggesting that the aphid circadian clock has evolved to adapt to aphid-specific needs (Cortés et al. 2010). In summary, the molecular clock network of the pea aphid shows similarities but also differences with respect to the *Drosophila* one.

### Photoperiodic receptors of aphids

While mammals, hymenopterans and many beetles have only CRY2, mosquitoes, butterflies and aphids have additionally the light-sensitive CRY1 (Deppisch et al. 2023). Cryptochromes are flavin-based photopigments that occur throughout all phyla. The light-sensitive animal cryptochromes have action spectra showing several peaks in the blue-light range, reduced sensitivity in the UV and no sensitivity at wavelengths beyond 500 nm (Fig. 4b) (Hoang et al. 2008). Thus, their action spectra resemble closely the short-wavelength part of the action spectrum recorded by Lees (1966, 1981) for the photoperiodic response of aphids (Fig. 4a), making them perfect candidates for photoperiod perception in addition to their role in circadian photoreception.

In addition, extraretinal opsins appear to contribute to photoperiodic sensitivity. In an attempt to localise the photoperiodic photoreceptor(s), Gao et al. (1999) performed a series of immunocytochemical experiments using a collection of vertebrate and *Drosophila* antibodies against different opsins, and identified a putative photoreceptor region in the aphid anterior ventral protocerebrum. Collantes-Alegre et al. (2018) identified and characterised the whole opsin repertoire present in the genome of the pea aphid and found that two opsins (Ap-C-Opsin and Ap-SWO4) are expressed in its lateral and dorsal brain and are thus suited to work as photoperiodic receptors. Moreover, opsin expression in aphid head extracts was shown to be much higher under short days as compared to long days (Collantes-Alegre et al. 2018) and this difference was only visible in an aphid strain that shows canonical photoperiodic responses (holocyclic strain) but no differences were observed in an aphid strain that lacks the capability to respond to photoperiodic changes (anholocyclic strain). This result supports a role of opsins in the photoperiodic time measurement, although it is not yet clear how the opsin containing cells could signal to the photoperiodic timer and the circadian clock.

### Location of the circadian clock and CRY1 in the aphid brain

Most interestingly, in situ hybridisation studies of *tim, per, cry1* and *cry2* genes show that they are expressed in neurons that are located in the lateral and dorsal (superior) protocerebrum of the aphid brain (Fig. 5a) (Barberà et al. 2017, 2022). According to the nomenclature used in *D. melanogaster*, these clock neurons were called lateral neurons (LN) and dorsal neurons (DN). In addition to the LN and DN, about seven *per* and per/*cry1* expressing neurons were found in the lamina (LaN) that have not been detected in *D. melanogaster* (Fig. 5a). These neurons appeared not to express *tim*, while two DNs that expressed *cry1* and *tim* did not express *per*. Immunocytochemistry with antibodies against PER and CRY1 confirmed the localisation of the molecular clock in the lateral and dorsal aphid brain (Colizzi et al. 2021) although some differences were observed to the in situ hybridisations (Fig. 5b). For example, the antibody against CRY1 did not stain any PER-negative DNs. As found in *D. melanogaster*, CRY1 co-localised with PER in about half of the clock neurons. All the CRY1-positive clock neurons showed daily PER/CRY1 oscillations of high amplitude. The CRY1 oscillations were highly synchronous in all neurons, suggesting that aphid CRY1, similarly to *Drosophila* CRY1, is light sensitive and its oscillations are synchronized by light–dark cycles. Nevertheless, in contrast to *Drosophila* CRY1, aphid CRY1 was not degraded by light, but steadily increased during the day and decreased during the night (Colizzi et al. 2021).

**Fig. 6:**
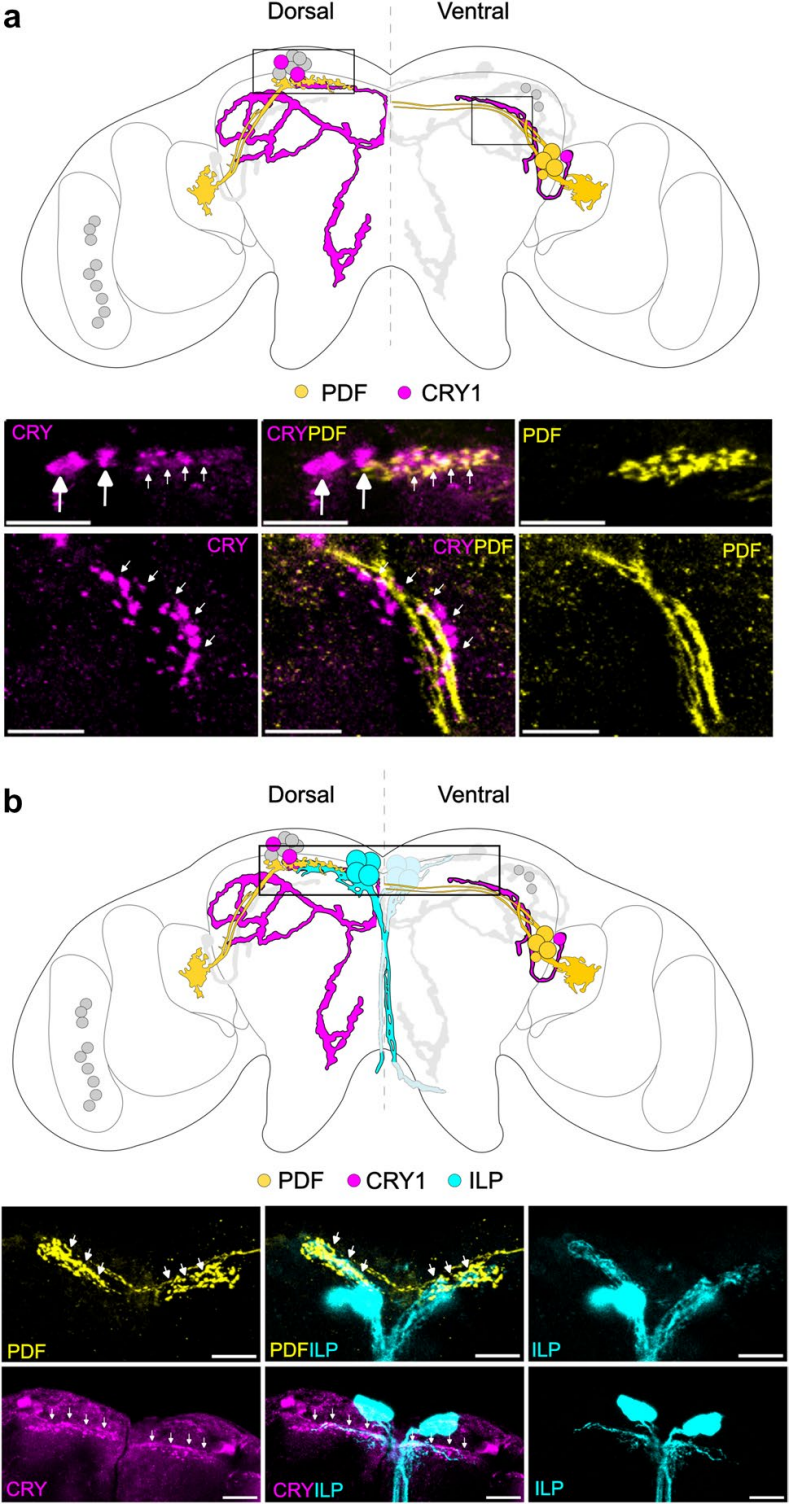
Interpretation of confocal images showing details of PDF, CRY1 and ILP4 arborisations. **a** Four PDF-positive cells per hemisphere project dorsally and contralaterally. Their arborisations are in close vicinity to the CRY1-positive arborisations in both the dorsal and lateral regions of the brain (rectangles). Confocal images of the contact regions are shown below (dorsal and lateral arborisations, top and bottom images, respectively). **b** ILP4-positive cells are in the pars intercerebralis and arborise both laterally in the pars lateralis (rectangle) and posteriorly to the corpora cardiaca. The lateral arborisations are near PDF- and CRY1-positive arborisations, suggesting a potential role in conveying photoperiodic signals from clock neurons to insulin-producing cells. Confocal images of the contact regions are also shown below. **a**, **b** The neuronal ramifications for CRY1-, PDF- and ILP4-positive cells, were created through 3D reconstructions of single neurons using Fiji software and then flattened for visualization. The colours of the final image were assigned using Inkscape software. All cells shown in colour and grey represent clock neurons. Scale bar: 20 µm

PER was always located in the nuclei of the clock neurons, while CRY1 was predominantly cytoplasmic and found in their neurites, revealing the projections of the PER/CRY1-positive neurons, except for those of the lamina (Colizzi et al. 2021). One PER/CRY1-positive clock neuron (called LN +) had its cell body in the lateral protocerebrum of each hemisphere and projected to the dorsal (superior) protocerebrum, where its terminals ended close to the dorsal clock neurons (Fig. 6a). This neuron strongly resembles the CRY1-positive ventrolateral neurons of the *D. melanogaster* clock. In the dorsal protocerebrum, two PER/CRY1-positive clock neurons were found that projected toward the *pars intercerebralis*. On the way, their arborisations overlapped with the dendrites of the four ILP4-positive neurons extending to the *pars lateralis* (Fig. 6b). This suggests that the PER/CRY1-positive DNs can transmit information about the photoperiod to the ILP4-positive neurosecretory cells which perfectly matches the old proposal by Steel and Lees (1977) of a photoperiodic photoreceptor and clock localised in the *pars lateralis* communicating photoperiod information to the NSC group I.

Once arrived medially in the *pars intercerebralis*, the CRY1-positive fibres turn posteriorly and run toward the thoracic nervous system. Before leaving the protocerebrum, at the height of the central complex, they give rise to a network of fibres in the lateral protocerebrum (Fig. 6). Overall, the arborisation pattern of the PER/CRY1-positive clock neurons shows similarities with the clock neurons and the output pathways of other insects (reviewed by Beer and Helfrich-Förster 2020). Only the fibres descending into the thoracic nervous system have so far not been described in other insects.

### The Pigment-Dispersing Factor (PDF) as putative output factor from the CRY1-negative clock neurons to the photoperiodic timer and effectors

The neuropeptide “Pigment-Dispersing Factor” (PDF) plays a pivotal role in the circadian clock of most insects investigated so far (reviewed in Helfrich-Förster 2009; Shafer and Yao 2014; Stengl and Arendt 2016; Beer and Helfrich-Förster 2020). In addition, PDF is implicated in photoperiodic timing of several insects (Shiga and Numata 2009; Meuti et al. 2015; Ojima et al. 2018; Nagy et al. 2019; Hasebe et al. 2022; Kotwica-Rolinska et al. 2022; Hidalgo et al. 2023). Nevertheless, PDF could not be identified in aphids by immunocytochemistry (Colizzi et al. 2021), and its encoding gene appeared also absent (Huybrechts et al. 2010). We hypothesized that the absence of PDF might be the reason for the weak circadian rhythmicity of pea aphids (Colizzi et al. 2021), but it remained questionable why this important peptide, which is present in virtually all panarthropods (Shafer and Yao 2014; Mayer et al. 2015) should be absent in the highly photoperiodic aphids.

Very recently, we found the reason for the apparent absence of PDF in aphids (Colizzi et al. 2023). Indeed, we finally identified the aphid PDF coding gene and showed that it shows significant differences to the so far known insect PDFs appearing to lack the 7 C-terminal amino acids that are typical for it. Nevertheless, its N-terminal sequence is well conserved and its expression pattern in the brain shows large similarities to that of other insect species leaving little doubt that it is indeed PDF.

With a newly generated aphid-specific PDF antibody, we stained four of the PER-positive lateral clock neurons in each hemisphere of the aphid brain (Fig. 6a). These clock neurons were CRY1-negative, but their projections overlapped with CRY1-positive fibres stemming from the one CRY1 neuron in the lateral brain and the two CRY1 neurons in the dorsal brain (Fig. 6a). In addition, the PDF-positive clock neurons projected to the *pars lateralis*, where they overlapped with dendrites of the insulin-like peptide (ILP) positive neurosecretory cells (Fig. 6b).

## Putative interplay between photoperiodic photoreceptors, circadian clock neurons and the neuroendocrine system controlling reproduction

**Fig. 7:**
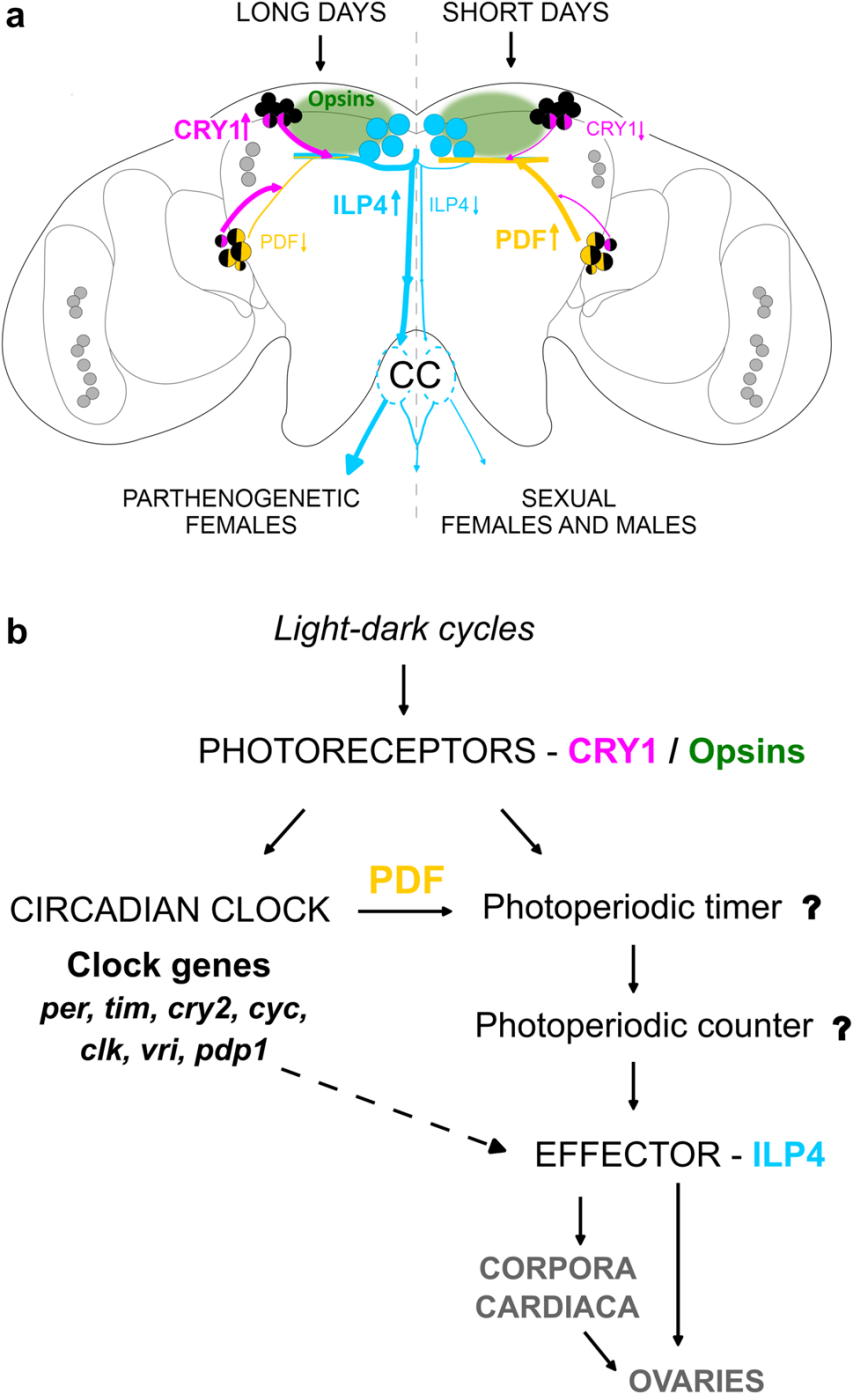
Putative interplay of CRY1, Opsins (Ap-C-Opsin and Ap-SWO4, after Collantes-Alegre 2018), PDF and ILP4 neurons in the control of photoperiodic reproduction. **a** Schematic representation of the aphid brain with the main molecular components involved in photoperiodism. The different mechanisms hypothesized in long and short days are depicted in the left and right hemisphere, respectively. Clock neurons that are thought to be involved in photoperiodism are coloured in black, while those that are not, are shown in grey. CC: *Corpora cardiaca* **b** Theoretical components of the photoperiodic system, already depicted in Fig. 2, with their putative molecular nature in aphids

In summary, the mentioned recent studies show that most components of the aphid photoperiodic system are in the superior (dorsal) protocerebrum of the brain, including the *pars lateralis* and *pars intercerebralis*; only some circadian clock neurons locate in the lateral brain (Fig. 7a).

Nevertheless, it is not so easy to separate the different components such as photoreceptors, circadian clock, photoperiodic timer, photoperiodic counter, and effectors. Of the photoreceptors, CRY1 is in a subset of the clock neurons and most probably used to synchronize the circadian clock to the light–dark cycles, in addition to transmitting photoperiod information to the proposed photoperiodic timer. Moreover, the fibres originating from the two CRY1-positive clock neurons in the dorsal brain largely overlap with the dendrites from the ILP4-positive neurosecretory cells, which are considered as effectors of the photoperiodic system. Thus, the photosensitive part of the circadian clock may directly transmit photoperiod information to the photoperiodic effectors.

Similarly, the PDF-positive circadian clock neurons could signal directly to the photoperiodic effectors instead of only signalling to the photoperiodic timer (Fig. 7b). The PDF terminals in the *pars lateralis* overlap with the putative dendrites of the ILP4 neurons, and *Pdf* expression and the length of the terminals have been shown to be modulated by photoperiod (Colizzi et al. 2023). On short days, *Pdf* expression increases, and PDF terminals lengthen, while on long days, *Pdf* expression is significantly lower and PDF terminals are shorter. PDF signalling to neurosecretory cells of the *pars intercerebralis* or *lateralis* has already been demonstrated in other insects (Shiga and Numata 2009; Nagy et al. 2019; Hidalgo et al. 2023), making it likely that this also occurs in aphids. PDF appears to promote the sexual reproductive mode of aphids (Colizzi et al. 2023) and the diapause mode of bugs in autumn (Kotwica-Rolinska et al. 2022), while it appears important for reproduction of *Drosophila* in spring and summer (Hidalgo et al. 2023).

These results suggest that the photoperiodic timer and counter are somehow integrated in the circadian and neurohormonal system. The photoperiodic timer might be in the photoreceptive dorsal circadian clock neurons themselves. These neurons strongly express *timeless* (Fig. 5a; Barberà et al. 2017), which has already been shown to be involved in the regulation of insect photoperiodic diapause (Bradshaw and Holzapfel 2007; see also Vaze et al. this issue). It is worth to recall that induction of the sexual phase is the way aphids enter diapause. TIM could even represent the photosensitive substance ‘c’ that accumulates in darkness and is degraded by light (via interaction with CRY1) as proposed for the ‘hourglass’ clock (Fig. 2b). If this is the case, these dorsal neurons perceive the external photoperiod directly and receive an additional circadian clock input from the CRY-negative PDF neurons. Thus, they can optimally integrate both inputs with each other. Alternatively, the photoperiodic timer may be in still unknown neurons that are in between the photoreceptor/circadian clock neurons and the ILP4 neurons. The photoperiodic counter, on the other hand could locate in the dendrites of the ILP4 neurons, which may sum up the inputs from photoreceptors and circadian clock neurons.

Future studies are necessary to reveal the exact nature and function of all photoperiodic components including the possible participation of other elements such as opsins (in the photoreception side) or melatonin, among others. Although much progress has been made in recent years, there are still many exciting questions about aphid photoperiodism that need to be answered by the next generation of insect scientists worldwide.
